# ATM and ATR Activities Maintain Replication Fork Integrity during SV40 Chromatin Replication

**DOI:** 10.1371/journal.ppat.1003283

**Published:** 2013-04-04

**Authors:** Gregory A. Sowd, Nancy Yan Li, Ellen Fanning

**Affiliations:** Department of Biological Sciences, Vanderbilt University, Vanderbilt Ingram Comprehensive Cancer Center, Nashville, Tennessee, United States of America; University of Michigan, United States of America

## Abstract

Mutation of DNA damage checkpoint signaling kinases ataxia telangiectasia-mutated (ATM) or ATM- and Rad3-related (ATR) results in genomic instability disorders. However, it is not well understood how the instability observed in these syndromes relates to DNA replication/repair defects and failed checkpoint control of cell cycling. As a simple model to address this question, we have studied SV40 chromatin replication in infected cells in the presence of inhibitors of ATM and ATR activities. Two-dimensional gel electrophoresis and southern blotting of SV40 chromatin replication products reveal that ATM activity prevents accumulation of unidirectional replication products, implying that ATM promotes repair of replication-associated double strand breaks. ATR activity alleviates breakage of a functional fork as it converges with a stalled fork. The results suggest that during SV40 chromatin replication, endogenous replication stress activates ATM and ATR signaling, orchestrating the assembly of genome maintenance machinery on viral replication intermediates.

## Author Summary

All cells have evolved pathways to maintain the integrity of the genetic information stored in their chromosomes. Endogenous and exogenous agents induce mutations and other damage in DNA, most frequently during DNA replication. Such DNA damage is under surveillance by a complex network of proteins that interact with one another to signal damage, arrest DNA replication, and restore genomic integrity before replication resumes. Many viruses that replicate in the nucleus of mammalian host cells have evolved to disable or evade this surveillance system, but others, e.g. polyomaviruses like SV40, activate it and somehow harness it to facilitate robust replication of viral progeny. We have sought to determine how SV40 induces and deploys host DNA damage signaling in infected cells to promote viral chromosome replication. Here we present evidence that, like host DNA, replicating viral DNA suffers damage that activates surveillance and repair pathways. Unlike host replication, viral DNA replication persists despite damage signaling, allowing defective replication products to accumulate. In the presence of host DNA damage signaling, these defective viral products attract proteins of the host damage surveillance network that correct the defects, thus maximizing viral propagation.

## Introduction

Faithful duplication of the genome is vital for cell proliferation. In metazoans, the consequences of inaccurate genome replication include cell death, premature aging syndromes, neuro-degeneration disorders, and susceptibility to cancer [1], [2]. The DNA damage signaling protein kinases ataxia telangiectasia-mutated (ATM) and ATM- and Rad3-related kinase (ATR), members of the phosphoinositide-3 kinase-like kinase (PIKK) family, act to ensure that cells with incompletely replicated or damaged DNA do not progress through the cell cycle [1]. ATM and DNA-dependent protein kinase (DNA-PK) respond primarily to DNA double strand breaks (DSB) that are associated with either Mre11/NBS1/Rad50 (MRN) [3] or Ku70/80 [4], respectively. Additionally, intracellular oxidation or alterations in chromatin structure can activate ATM kinase [5], [6]. In contrast, single-stranded DNA (ssDNA) bound by RPA activate ATR [7], [8]. When activated, ATM and ATR phosphorylate consensus SQ/TQ motifs in target proteins at sites of damage, e.g. the histone H2AX, which facilitates recruitment of repair proteins and activation of downstream kinases Chk1 and Chk2 that enforce the checkpoint [8], [9].

Failure to activate DNA damage checkpoints results in genome instability syndromes. Mutations in the human ATM gene can cause the cancer-prone disorder ataxia telangiectasia. Hypomorphic mutations in the ATR gene can cause the genomic instability disorder Seckel Syndrome, but complete loss of ATR results in cell death [10], [11]. The central roles of ATM and ATR in genome maintenance suggest the potential to manipulate their activity for cancer chemotherapy, fueling the development of potent small molecules that specifically inhibit ATM and ATR activities *in cellulo* [12], [13].

Interestingly, multiple animal viruses have evolved to manipulate DNA damage signaling pathways to facilitate viral propagation [14]. Some viruses, e.g. Herpes simplex, evade or disable DNA damage response pathways that result in inappropriate processing of viral DNA [15], [16]. In other cases, viral infection appears to activate checkpoint signaling and harness it to promote the infection. HIV, human papillomaviruses, and polyomaviruses induce and depend on ATM signaling for viral propagation [17], [18], [19], [20], [21], [22]. However, mechanistic understanding of how these viruses activate damage signaling and exploit it for viral propagation is limited.

Simian Virus 40 (SV40), a polyomavirus that propagates in monkey kidney cells, has served as a powerful model to study eukaryotic replication proteins and mechanisms *in vivo* and *in vitro* [23], [24], [25], [26], [27]. Checkpoint signaling proteins are dispensable for SV40 DNA replication *in vitro*, yet in infected cells, ATM or ATR knockdown, over-expression of kinase-dead variant proteins, or chemical inhibition of checkpoint signaling clearly decreases or delays SV40 chromatin replication [26], [28], [29], [30]. To determine how checkpoint signaling facilitates viral replication in SV40-infected primate cells, we have utilized small molecule inhibitors of the PIKK family members ATM, ATR, and DNA-PK to suppress checkpoint signaling in host cells during three specific time windows after SV40 infection. Characterization of the resulting viral DNA replication products reveals that inhibition of ATM or ATR, but not DNA-PK, reduced the yield of unit length viral replication products and caused aberrant viral DNA species to accumulate. ATM inhibition led to unidirectional SV40 DNA replication and concatemeric products, whereas ATR inhibition markedly increased broken SV40 DNA replication forks. Our results strongly suggest that unperturbed viral chromatin replication in infected cells results in double strand breaks, activating checkpoint signaling and fork repair to generate unit length viral replication products.

## Results

### SV40 chromatin replication activates DNA damage signaling

Replicating SV40 chromatin in infected cells has been visualized by fluorescence microscopy in prominent subnuclear foci that co-localize with Tag and several host proteins essential for viral DNA replication *in vitro*, suggesting that these foci may represent viral chromatin replication centers [26], [29], [31]. However, SV40 infection activates ATM and ATR signaling, and several DNA damage signaling proteins, e.g. MRN, γH2AX, ATRIP, and 53BP1, co-localize with Tag in these foci [28], [29], [30], [32], implying a link between SV40 replication and damage signaling. On the other hand, interaction of ectopically expressed Tag with the spindle checkpoint protein Bub1 can also induce cellular chromosome breaks [33], indicating that Tag interference with host mitotic checkpoint proteins may suffice to damage genomic DNA in uninfected cells.

**Figure 1:**
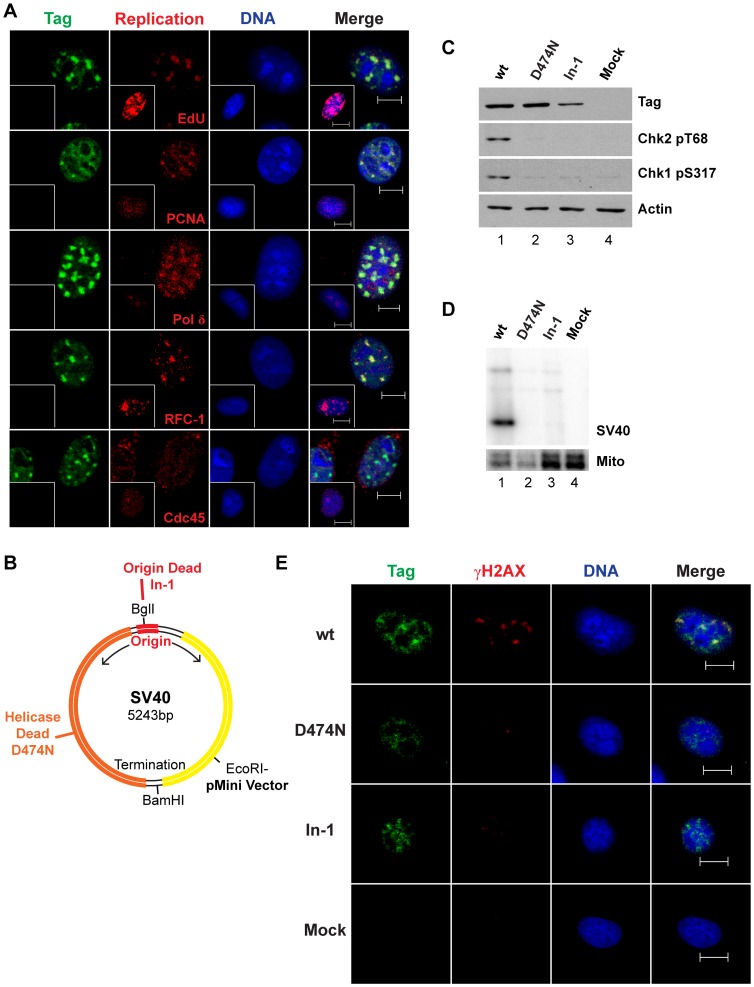
SV40 chromatin replication results in DNA damage signaling. (A) Representative images of chromatin-bound Tag and host DNA replication proteins in SV40- and mock-infected (inset) BSC40 cells at 48 hpi. (B) Features of the SV40 genome and the insertion site of pMini vector [34]. Mutation of Tag residue 474 from D to N abrogates helicase activity [34]. The defective SV40 origin mutant, In-1, features an insertion of a single GC bp in the center of the viral origin allowing Tag binding, but not origin activation [35]. (C, D, E) BSC40 cells transfected with the indicated pMini SV40 plasmids were analyzed by (C) western blot after 24 h, (D) Southern blot of low molecular weight DNA after 48 h [34], [73], or (E) immunofluorescence microscopy of chromatin-bound proteins. In (D), SV40 or Mitochondrial probe signal is denoted by SV40 or Mito, respectively. Scale bars in (A) and (E), 10 µm.

As a first step to assess a potential link between SV40 chromatin replication and DNA damage signaling, viral replication centers in SV40-infected BSC40 monkey cells were characterized in detail. Chromatin-bound Tag was visualized in subnuclear foci as expected and colocalized with newly replicated DNA that had incorporated the deoxynucleoside EdU (Figures 1A and S1A). Chromatin-bound PCNA, DNA polymerase δ, and the clamp-loader RFC, host proteins that are essential for viral DNA replication *in vitro*, colocalized with Tag foci in both BSC40 and human U2OS cells at 48 hours post infection (hpi) (Figures 1A, S1B–D, and S2). In contrast, Cdc45, an essential component of the CMG host replicative helicase that colocalized with replicating chromatin in mock-infected U2OS cells (Figure S2C, D), was virtually excluded from viral replication centers (Figures 1A, S1E, and S2C, D). The results strongly suggest that in infected cells, these chromatin-bound Tag foci represent sites of viral, rather than host, chromatin replication.

We next asked whether SV40 DNA replication itself might induce DNA damage signaling in the absence of viral infection. Toward this end, the plasmids pMini SV40-wt, and its replication-defective variants lacking Tag helicase activity (D474N) [34], or containing a single base pair insertion that inactivates the viral origin (In-1) [35], were transfected into BSC40 monkey cells (Figure 1B). As expected, all three plasmids expressed Tag, but only the SV40-wt plasmid replicated (Figure 1C, D). SV40-wt activated phosphorylation of Chk1 and Chk2 more robustly than either of the replication-defective constructs (Figure 1C, compare lane 1 to lanes 2–3). Moreover, prominent γH2AX foci, a marker of DNA damage signaling in chromatin [36], colocalized with chromatin-bound Tag in viral replication centers in SV40-wt transfected cells (Figure 1E). In contrast, the few γH2AX foci detected in cells transfected with the replication defective plasmids did not colocalize with Tag. Thus, in the context of transfected cells, viral DNA replication, but not SV40-driven Tag expression, is sufficient to induce DNA damage signaling, suggesting that DNA breaks in replicating viral chromatin may activate checkpoint signaling.

### Inhibition of ATM disrupts viral DNA replication centers

**Figure 2:**
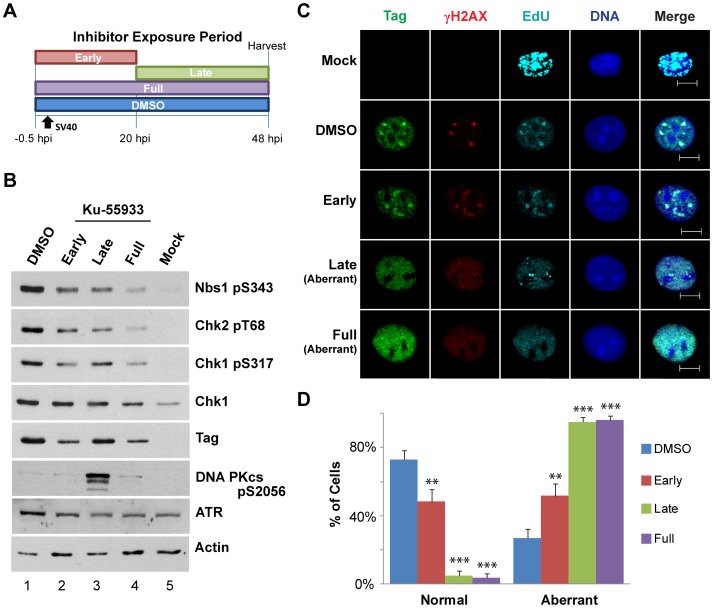
ATM inhibition during viral DNA replication disrupts viral replication centers. (A) Experimental scheme for treatment of cells with inhibitor during phases of a 48 h SV40 infection. Early: inhibitor present from −0.5 to 20 hpi. Late: inhibitor present from 20 to 48 hpi. DMSO and Full: solvent or inhibitor, respectively, present from −0.5 to 48 hpi. (B) Western blot of cells treated with Ku-55933 as described in (A). (C) Immunofluorescence of cells treated with Ku-55933 as described in (A) and fixed at 48 hpi. Scale bars, 10 µm. (D) Tag staining patterns, as in (C), were quantified. Graph shows the average of 3 independent experiments.

To determine the temporal requirements for ATM activity during infection, we exposed infected cells to the specific ATM chemical inhibitor Ku-55933 [12] during the early phase (virus entry, Tag expression, host DNA synthesis), late phase (viral DNA replication, late gene expression, and virion assembly), or throughout a 48-hour infection (Figure 2A). Infected cells exposed to the Ku-55933 solvent, DMSO, served as a positive control. Mock-infected cells not treated with inhibitor served as a negative control. ATM activity was stimulated by infection, as indicated by phosphorylated Nbs1 and Chk2 in western blots (Figure 2B, compare lane 1 to lane 5), reduced by the presence of Ku-55933 in either the early or late phase of infection (Figure 2B, compare lanes 2, 3 to lane 1), and nearly abolished by the presence of Ku-55933 throughout infection (Figure 2B, lane 4).

To assess the impact of ATM inhibition during each phase of infection on viral chromatin replication, we visualized viral replication centers and DNA damage signaling in each infected cell population using immunofluorescence microscopy (Figure 2C). In infected cells exposed to DMSO, the normal, brightly stained viral replication centers with colocalized Tag, EdU, and γH2AX were observed (Figure 2C). When Ku-55933 was present only during the early phase of infection, about half of the cells displayed normal replication centers with colocalized Tag, EdU and γH2AX foci (Figure 2C and D). However, aberrant pan-nuclear staining of Tag, EdU, and γH2AX predominated when Ku-55933 was present during the late phase or throughout infection (Figure 2C and D). Taken together, the results demonstrate that ATM activity was beneficial but not essential during the early phase of infection, whereas it was vital for the assembly and/or stability of viral replication centers during the late phase of infection.

### Inhibition of ATM activity reduces the quantity and quality of viral replication products

**Figure 3:**
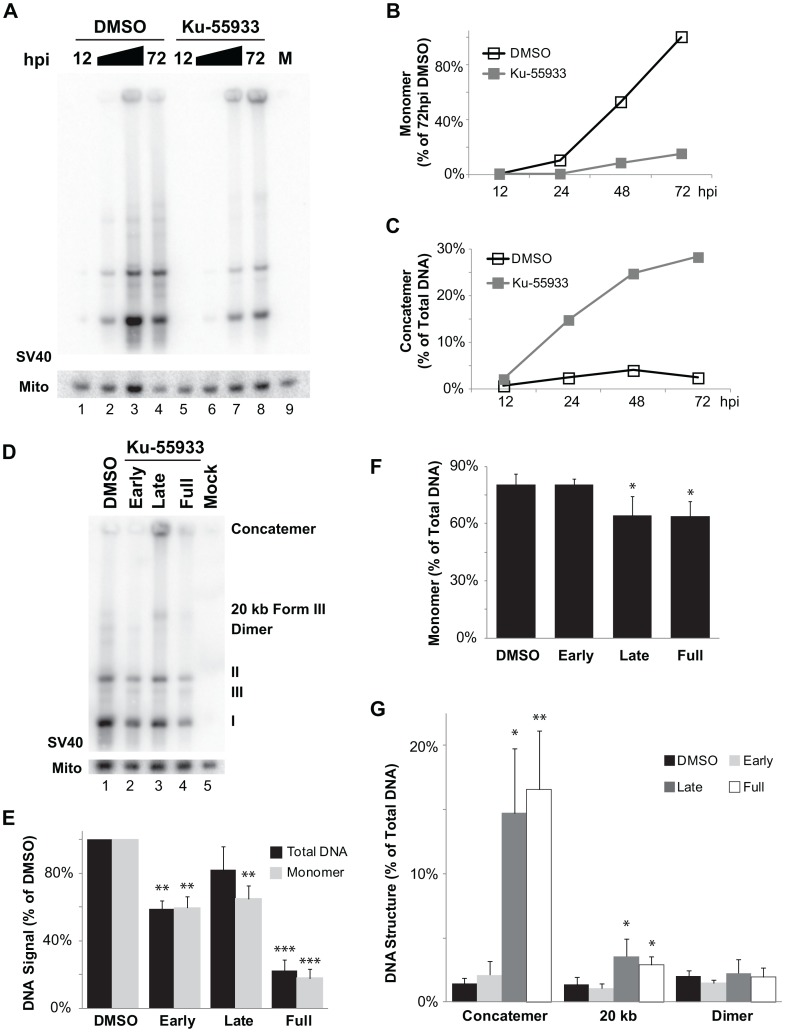
Ku-55933 treatment during viral DNA replication increases aberrant DNA structures. (A) Southern blot of DNA from SV40 infected BSC40 cells in the presence of DMSO or Ku-55933. DMSO or Ku-55933 was present from 30 min prior to infection until cell collection timepoint. M represents Mock-infected cells. (B) Each normalized monomer SV40 form I, II, and III product in (A) was graphed as a fraction of the corresponding normalized monomer produced at 72 hpi in the DMSO control infection. (C) Graph of the percentage of DNA products represented by concatemers in panel (A). (D) Southern blot of SV40 DNA replicated in the presence of Ku-55933 during phases of a 48 h infection in BSC40 cells as explained in Figure 2A. (E) Quantification of total and monomeric SV40 DNA signal normalized to DMSO control from southern blots as in (D). (F, G) Graph of DNA structures (monomer: F and DNA Structure: G) accumulating on southern blots as in (D). Graphs in (E–G) represent 3 to 4 independent experiments.

The links between ATM activity and SV40 replication centers led us to hypothesize that inhibition of ATM might affect not only the level, but perhaps also the nature of the viral DNA replication products. To investigate this possibility, we used southern blotting to analyze total intracellular DNA from SV40-infected BSC40 cells that had been treated with DMSO or Ku-55933 throughout infection (Figure 3A). Inhibition of ATM reduced the level of 5.2 kbp viral DNA products migrating as form I (supercoiled), form II (nicked), and form III (linear), relative to that in the DMSO-treated control infections (Figure 3A, compare lanes 1–4 to 5–8). However, ATM inhibition also caused accumulation of high molecular weight SV40 DNA products too large to enter the gel (Figure 3A, compare lanes 3, 4 to lanes 7, 8). These large products failed to migrate into the gel after restriction digestion with enzymes that cut host DNA but not SV40 DNA. In contrast, most of these products collapsed into unit length linear SV40 DNA after digestion with an enzyme that cleaves SV40 DNA once (Figure S3A), indicating that the large DNA products contain head-to-tail repeats of unit length viral DNA.

To quantify the data in Figure 3A, the signal in SV40 monomer bands (forms I, II, and III) in each sample was normalized to that of mitochondrial DNA (Mito) in the same sample. This normalized monomer signal in each sample was then compared to that of the normalized monomer bands in the positive control at 72 hpi. (Figure 3A, lane 4) and graphed in Figure 3B. The graph reveals that ATM inhibition reduced unit length SV40 product by at least 5-fold compared to the DMSO control infections (Figure 3B). Quantification of the concatemeric SV40 DNA in each sample relative to that of the total SV40 signal in the same sample revealed that ATM inhibition increased accumulation of viral DNA concatemers by an order of magnitude compared to that in the DMSO control samples (Figure 3C). Thus, inhibition of ATM throughout infection reduced monomeric and increased concatemeric SV40 DNA products.

To determine what stage of SV40 infection required ATM activity, total intracellular DNA was extracted from infected BSC40 cells exposed to Ku-55933 during three time windows, as diagrammed in Figure 2A. The purified DNA was separated by gel electrophoresis and analyzed in southern blots (Figure 3D). Inhibition of ATM either early or throughout infection reproducibly reduced the level of total viral DNA and monomeric DNA products by 50–80% relative to that generated in the DMSO-treated control infection (Figure 3D, E). Similarly, in the late phase of infection, inhibition reduced viral DNA monomers to a level comparable to that observed when ATM was inhibited during the early phase, yet total viral DNA was only insignificantly decreased compared to DMSO-treated cells (Figure 3D, E). SV40 monomers comprised about 80% of the total viral DNA signal in samples from infected cells exposed to DMSO or Ku-55933 during early phase (Figure 3F). In contrast, monomers comprised only 64% of the total signal in samples treated with Ku-55933 late or throughout infection (Figure 3F). When Ku-55933 was applied either during the late phase or throughout infection, the fraction of total viral DNA in concatemers increased 10- and 11-fold, respectively, relative to the fraction in DMSO-treated infected cells (Figure 3G). The fraction of total SV40 DNA migrating at 20-kbp linear also increased in cells treated with Ku-55933 late or throughout infection, relative to that in DMSO-treated control infections (Figure 3G).

To confirm these findings in a different cell background, the temporal requirements for ATM activity were also determined in SV40-infected human U2OS cells, with similar results (Figure S3B–E). Taking the results together, we infer that SV40-infected cells require ATM signaling, primarily during the late phase of infection, to favor production of unit-length genomes rather than aberrant products.

### ATM inhibition increases rolling circle DNA replication and strand invasion

**Figure 4:**
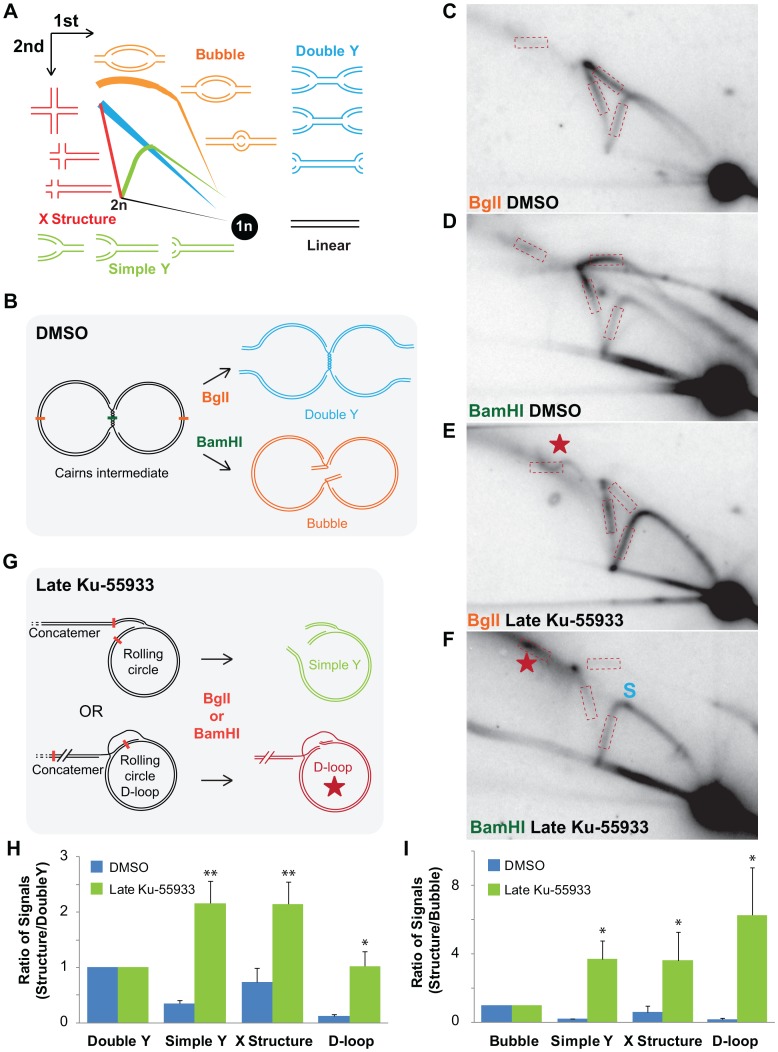
ATM inhibition increases recombination and unidirectional replication. (A) Diagram of neutral 2 d gel electrophoresis arcs generated from digested SV40 DNA. (B) Replicating viral DNA extracted from unperturbed SV40-infected cells consists primarily of circular, late replication intermediates called late Cairns intermediates. Digestion of late Cairns intermediates with BglI yields large double Ys, whereas BamHI digestion yields large bubbles. (C, D, E, F) Southern blot of neutral 2 d gel of BglI-cleaved DNA replicated in the presence of DMSO (C, D) or Ku-55933 (E, F) during the late phase of a 48 h SV40 infection in BSC40 cells. DNA was cleaved within the viral origin of replication with BglI (C, E) or the region of fork convergence with BamHI (D, F). The red star denotes an arc representing strand invasion events (D-loops) or highly branched molecules [38], [39]. On the simple Y arc in (F), S denotes a replication stall point near the viral origin of replication. Dashed boxes denote regions of each arc quantified in (H) and (I). (G) Concatemers of SV40 DNA that accumulated when ATM was inhibited can arise by either replication- (top) or recombination- (bottom) dependent rolling circle replication. Digestion of replication-dependent rolling circles with BglI or BamHI results in simple Ys of all sizes. Digestion of recombination-dependent rolling circles creates D-loops of all sizes. (H) Graph of DNA signal present on simple Y, double Y, X structure, or D-loop arc divided by DNA signal in the double Y arc from DNA digested with BglI. (I) Graph of DNA signal from BamHI digested DNA in simple Y, bubble, X structure, or D-loop arc divided by DNA signaling in the bubble arc. Each graph in (H) and (I) represents the average of 3 to 4 independent experiments.

To better understand how the aberrant viral replication products arise, we compared replication intermediates generated with and without Ku-55933 during the late phase of infection. The total DNA was first digested with a restriction nuclease that cleaves SV40 once in the viral origin (BglI) or once in the region of termination (BamHI). Neutral two-dimensional (2 d) gel electrophoresis was then used to separate viral replication intermediates from the accumulated non-replicating unit-mass SV40 DNA, followed by southern blotting using the whole SV40 genome as the probe [37]. Replicating viral DNA is present in the form of circular, converging forks known as Cairns intermediates (Figure 4B). The digestion of Cairns intermediates with BglI or BamHI results in double Ys or bubbles, respectively (Figure 4A, B). In the BglI-cleaved DNA from DMSO-treated control infections, the bubble arc was absent and the unit-mass viral DNA migrated in the 1 n spot as expected (Figure 4A–C). Also as expected, an intense double Y arc indicative of converging forks and an X structure signal indicative of hemi-catenates or Holliday junctions were observed (Figure 4C). In addition, the simple Y arc signal revealed some unidirectional replicating forks (Figure 4C) that can be most easily explained by rolling circle replication. When BamHI-cleaved DNA from DMSO-treated infected cells was analyzed by 2 d gel electrophoresis, the bubble arc was detected and the double Y arc was absent, as expected (Figure 4D). Similar to BglI digestion, both an X structure and a weaker simple Y arc were present (Figure 4D).

In contrast, the pattern of BglI-digested viral replication intermediates generated in the presence of Ku-55933 displayed a much fainter double Y arc and a more intense simple Y arc (compare Figure 4E with C). Similarly, X structures and D-loops, or other complex branched intermediates (red star), were more prominent when ATM was inhibited (compare Figure 4E with C), consistent with increased Holliday junction formation between replicating rolling circles [38], [39]. Likewise, BamHI-cleaved replication intermediates from Ku-55933-treated infections displayed a robust simple Y arc and a corresponding decrease in the bubble arc (Figure 4F). Moreover, the intense X structure and D-loop arcs were retained (Figure 4F). These patterns suggest that inhibition of ATM sharply increased the frequency of rolling circle replication (Figure 4G). Quantification of the signal present in the simple Y, X structure, D-loop, and double Y arcs from BglI-digested DNA (Figure 4C, E boxes) showed that ATM inhibition increased the abundance of simple Ys, X structures, and D-loop arcs relative to the double Y arc by six, three, and eight-fold, respectively, from three to four independent experiments (Figure 4H). Analogously, quantification of BamHI-digested DNA (Figure 4D, F boxes) revealed ATM inhibition increased the quantities of simple Ys, X structures, and D-loop arcs relative to the bubble arc (Figure 4I). We conclude that the ATM inhibitor Ku-55933 increased both rolling circle replication and strand invasion events at the expense of bidirectional SV40 chromatin replication.

### Caffeine inhibits SV40 chromatin replication

The importance of ATM activity in SV40 chromatin replication suggested the possibility that other checkpoint kinases might also contribute to viral infection. To further explore this question, we treated SV40-infected BSC40 cells with caffeine, a less selective inhibitor of both ATM and ATR *in vitro* and of the S/G2 checkpoints *in vivo* [40]. Of note, caffeine is structurally unrelated to the more potent Ku-55933 and ATR inhibitors [12], [13]. As expected, caffeine inhibited phosphorylation of Chk1 and Chk2 when present during the late phase or throughout infection (Figure S4A, B) but also hyper-activated DNA-PK (Figure S4B, compare lane 1 with lanes 2–4) [41]. Caffeine reduced the level of total viral DNA products in SV40-infected BSC40 cells to less than 1% of the control level when caffeine was present throughout infection (Figure S5A, B). Exposure to caffeine late or throughout infection reduced the fraction of total viral DNA signal in monomers (form I, II, III) and increased the fraction in concatemers and other aberrant products (Figure S5A, C, D). Similarly, in SV40-infected U2OS cells, caffeine reduced total viral replication products and increased the fraction of aberrant products (Figure S5E–H). The results further confirm the role of ATM activity in SV40 chromatin replication in infected cells and suggest that ATR and/or DNA-PK activity may stimulate viral replication.

### DNA-PK activity is dispensable for SV40 chromatin replication

Although SV40 infection did not activate DNA-PK, it was activated in infected cells exposed to Ku-55933 or caffeine, as evidenced by DNA break-dependent auto-phosphorylation of DNA-PK at S2056 [41] (Figures 2B, S4B). To test for a potential role of DNA-PK activity in viral chromatin replication, SV40-infected BSC40 cells were exposed to small molecule inhibitors of DNA-PK during the early or late phase, or throughout infection and total intracellular DNA was analyzed by southern blotting (Figure S6A–C). When DNA-PK was inhibited with either Nu7441 or Nu7026, the levels of viral monomer and aberrant viral DNA products closely resembled those in SV40-infected BSC40 cells (Figure S6D). Moreover, inhibition of DNA-PK had little or no effect on viral replication centers (data not shown). Thus, it is unlikely that DNA-PK has a major role in viral chromatin replication in unperturbed infected cells.

### ATR inhibition decreases SV40 DNA replication

The role of ATR kinase activity in infection was directly examined by treating SV40-infected BSC40 cells with a specific small molecule inhibitor of ATR, VE-821 (ATRi) [13], during three different time windows of infection (Figure S7A). As expected, ATRi caused a third of the cells to lose viability over 48 h, but SV40-infected and mock-infected cells were equally sensitive (Figure S7B). SV40 infection activated Chk1, as indicated by phosphorylation of Ser317 (Figure S7C, compare lane 1 with lane 5), and ATRi effectively suppressed ATR activation during each time window (Figure S7C, lanes 2–4).

**Figure 5:**
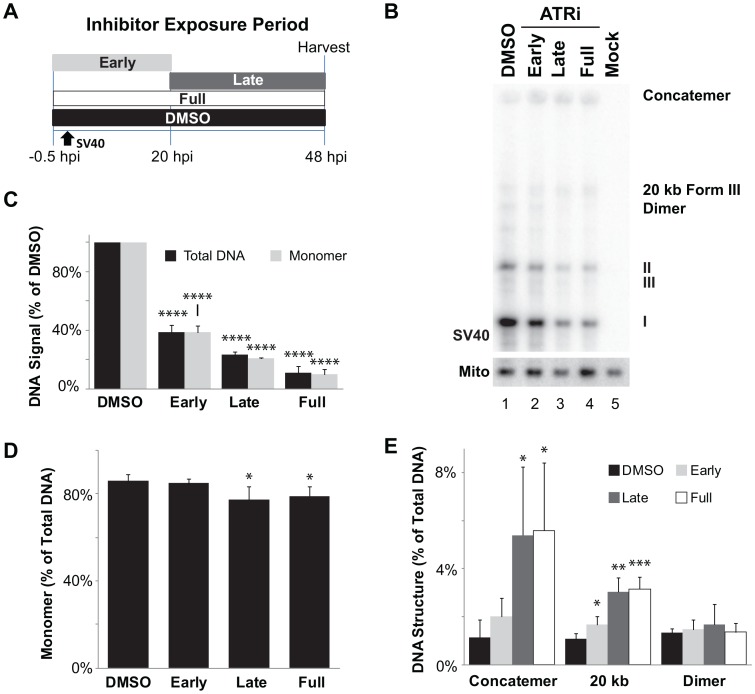
ATR is crucial for SV40 chromatin replication. (A) Scheme for application of ATRi during phases of a 48 h SV40 infection. (B) Southern blot of DNA replicated in BSC40 cells when ATRi was present during phases of a 48 h SV40 infection described in (A). (C) Graph of total viral or SV40 monomer DNA signals normalized to SV40 DNA replicated in the presence of DMSO from southern blots as shown in (B). (D, E) Graph of of monomer (D) or aberrant (E) structure(s) accumulated as a result of ATR inhibition from southern blots as shown in (C). Each bar in (C–E) shows the average from 3 to 4 independent experiments.

Viral DNA replication products from the four cell populations and mock-infected cells were analyzed by southern blotting and quantified relative to mitochondrial DNA in the same samples. In the presence of ATRi, the level of total viral DNA replication products declined markedly relative to that in DMSO-treated control infections, amounting to only 10% of the control when ATRi was present for the full 48 h (Figure 5B, C). In cells exposed to ATRi during the late phase or throughout infection, the fraction of viral DNA products in monomers (forms I, II and III) dropped, whereas that in concatemers and other aberrant products rose (Figure 5B–E and Figure S8A). Analysis of viral replication products from SV40-infected U2OS cells exposed to ATRi demonstrated a similar requirement for ATR activity (Figure S8B–D). Taken together, these results indicate that infected cells require ATR activity before, as well as during viral chromatin replication, for normal accumulation of viral genomes.

### Broken and/or stalled forks accumulate in ATR-inhibited SV40-infected cells

**Figure 6:**
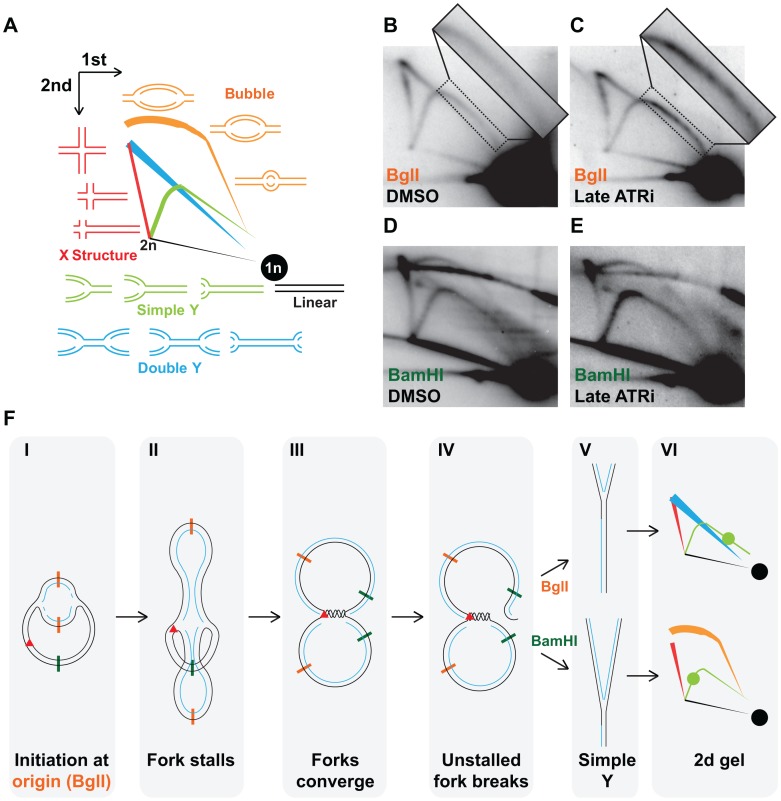
ATR inhibition results in fork stalling and breakage of converging forks. (A) Schematic of replication intermediate migration patter on a neutral 2 d gel generated from digested SV40 DNA. (B, C, D, E) Southern blot of neutral 2 d gel electrophoresis of BglI- (B, C) or BamHI-cut (D, E) DNA from SV40-infected BSC40 cells exposed to DMSO (B, D) or ATRi (C, E) during the late phase of SV40 infection as described in Figure 5A. (F) Diagrams of replication intermediates on a simple Y arc produced when ATR was inhibited. BamHI (green) and BglI (orange) sites are denoted by colored lines. I. Replication initiates at the origin and proceeds bidirectionally producing theta replication intermediates. II. Replisomes continue replication until one encounters a replication block (red triangle) causing one stalled fork. III. The stalled replication fork is closest to orange BglI site (viral origin of replication). The functional replisome continues replication and converges with the stalled replication fork. IV. One-sided DSB forms at the replicating fork of late Cairns intermediate shown in (III) as it translocates toward the stall site. V. Simple Y created by digestion of the broken late Cairns intermediate shown in (IV) with BglI or BamHI. VI. Diagram of the predicted outcome of the simple Y shown in panel (V) following neutral 2 d gel electrophoresis and southern blotting. The stall point on the simple Y arc (light green circle) corresponds to the simple Y in panel (V).

The structures of viral replication intermediates generated in the presence and absence of ATR kinase activity were characterized by using neutral 2 d gel electrophoresis and southern blotting. As expected, BglI-digested SV40 replication intermediates from control infections displayed a strong double Y arc indicative of converging forks, X structures, and a weaker simple Y arc with both legs of similar intensity (Figure 6B). In contrast, BglI-digested replication intermediates from ATRi-treated cells yielded a novel pattern (Figure 6C). Although the double Y and X structure arcs closely resembled those in the DMSO control, the simple Y arc displayed much greater intensity in the leg closer to the 1 n linear DNA (Figure 6B and C, zoomed box) than in the other leg closer to 2 n linear DNA. This pattern is not consistent with rolling circle replication, which generates a uniformly intense simple Y arc (Figure 4) or with two stalled replication forks, of which one breaks, creating an asymmetric simple Y [42]. The observed pattern is also inconsistent with one normal replication fork and one slower moving fork, which would converge asymmetrically to generate a cone-shaped signal between the X structure arc and the Y arc [43]. However, the novel pattern observed could arise if one fork stalls prematurely (Figure 6F, I, II), while the other fork progresses until it encounters the stalled fork and then breaks, generating a broken late Cairns intermediate (Figure 6F, III, IV) [37]. Close inspection of the intense leg of the Y arc reveals that its intensity is uneven, suggesting that it may arise from a series of closely spaced break sites along the Y arc (Figure 6C). If the break sites reside 2.5 kb or less from the BglI cleavage site, the intensity of signals would be greater in the right leg of the simple Y arc, as observed (Figure 6C, box). This interpretation predicts that if replication products from the ATRi-treated infection were digested with BamHI, which cleaves 2.5 kb from the BglI site, the sites of breakage, and hence greater signal intensity, should shift to the left leg of the simple Y arc, closer to the 2 n linear DNA (Figure 6A, E). Indeed, this shift was observed (compare Figure 6D with E), confirming that when the moving replication fork encountered a fork that had stalled in the presence of ATRi, the moving fork broke (Figures 6F and S9).

## Discussion

**Figure 7:**
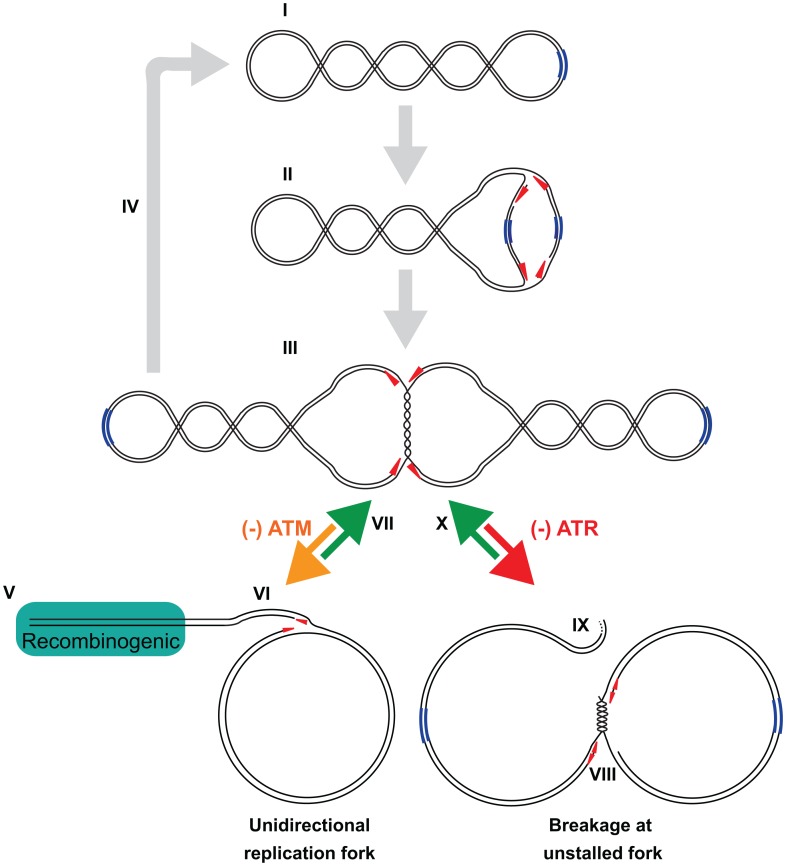
Model of ATM and ATR functions in SV40 DNA replication. (I) Tag initiates viral DNA replication at the viral origin of replication (blue) and the two replication forks progress bidirectionally (red arrowheads). For simplicity, proteins are not shown. (II) Viral DNA replicates quickly until the forks converge to form a late Cairns intermediate (III), which slowly completes replication. (IV) Topoisomerase IIα decatenates fully replicated DNA molecules, yielding two form I daughter molecules. (V) When ATM is inhibited, a one-ended double strand break at a replication fork leads to loss of the replication machinery, while the other fork continues to replicate DNA, generating a rolling circle (VI). (VII) ATM kinase activity facilitates the repair of one-ended double strand breaks. (VIII) When ATR is inhibited, a stalled replication fork remains stable until a functional replication fork approaches it, generating a broken replication intermediate (IX). (X) ATR kinase activity facilitates convergence of moving fork with the stalled fork. We suggest that in the presence of ATM and ATR, repair proteins act on the defective intermediates V and IX to reassemble an intermediate with two functional forks.

This study presents several lines of evidence that SV40 harnesses host DNA damage signaling for quality control of viral chromatin replication. We show that viral DNA replication *in vivo* is sufficient to induce DNA damage signaling at viral replication centers (Figures 1, S1, S2), suggesting that DNA lesions may arise in unperturbed replicating viral DNA. Importantly, damage signaling is vital to maintain viral replication centers (Figures 1, 2). Furthermore, suppression of ATM and/or ATR signaling increases the level of aberrant viral replication products at the expense of unit length viral DNA (Figures 3–5, S3, S5, S8), implying that viral replication-associated damage in infected cells requires ATM and ATR signaling to promote repair of viral replication forks. Lastly, our results indicate that the defective replication intermediates resulting from inhibition of ATM (Figure 4) and ATR (Figures 6, S9) are distinctive. Taken together, our results support a model in which ATM and ATR serve different but complementary roles in orchestrating repair at viral replication forks (Figure 7).

### DNA damage signaling nucleates the assembly of SV40 replication centers

SV40 chromatin replication centers resemble over-sized host DNA damage response foci (for a comparison, see Figure 1 in ref [29]), where diverse damage signaling and DNA repair proteins assemble on chromatin at a DNA lesion and dissociate when repair is completed [1], [44]. Many of the same signaling and repair proteins are found at both viral replication centers and host damage response foci [18], [21], [22], [28], [29], [30], [32], [33] (Sowd, unpublished). However, unlike the prominent viral replication centers, the punctate host damage response foci encompass megabase regions of chromatin, raising the question of how SV40 mini-chromosomes give rise to the large subnuclear foci observed in the microscope. The size of SV40 replication centers increases with the number of incoming viral genomes and with time post-infection in permissive primate cells [29], suggesting that our ability to detect viral replication centers depends on the ability of each infected cell to generate 10–100 thousand daughter genomes [45]. Moreover, unperturbed viral replication centers display nascent ssDNA (Sowd, unpublished) and DNA breaks that are likely responsible for activating checkpoint signaling, analogous to lesions that nucleate host damage response foci.

A major difference between SV40 replication centers and host damage response foci is that checkpoint signaling does not inhibit the viral replication machinery, whereas Chk2 phosphorylation of the purified host replicative helicase Cdc45/Mcm2-7/GINS inhibits its helicase activity *in vitro* [46] and Chk1 inhibits Cdc45 recruitment to chromatin to initiate replication *in vivo* [47]. Based on these considerations, we suggest that SV40 replication centers serve as hubs where host replication and repair factors efficiently service many client viral genomes in close proximity. These hubs are nucleated and maintained by the assembly of the ATM and ATR signaling complexes at sites of viral replication stress, followed by recruitment of downstream repair factors [1]. Of note, all of the host proteins needed for SV40 DNA replication *in vitro* [23], [24], [25] also function in host DNA repair [23], [25], [48], [49]. Thus SV40, though it encodes only a single essential replication protein, has evolved a rather remarkable strategy to generate viral replication compartments.

### ATM signaling orchestrates reassembly of viral replication forks, reducing unidirectional replication forks

Recent studies in several laboratories, including ours, established that knockdown or inhibition of ATM in polyomavirus-infected cells reduced production of unit length viral genomes [21], [22], [28], [29]. Since these studies evaluated only unit length viral DNA, the aberrant viral replication products generated by unidirectional replication forks were overlooked (Figures 3, 4, S3). Interestingly, total intracellular DNA from unperturbed infected CV1P cells has also been reported to contain head-to-tail SV40 DNA repeats of 50 to 100 kbp at very late times after infection [45]. These observations indicate that concatemers may be a normal product of viral replication, and suggests that inhibition of ATM activity might simply increase the frequency of unidirectional replication, advance its timing, or both.

Although replication-associated breaks may be a rare event during unperturbed viral DNA replication, the large number of replicating viral genomes would facilitate their detection, particularly when ATM activity is suppressed. Yet surprisingly, when undigested total intracellular DNA from an ATM-inhibited infection was analyzed by 2 d gel electrophoresis, bidirectional replication was still observed (data not shown) and unit length viral DNA remained the predominant product when ATM was inhibited (Figures 3 and S3). These observations can be most simply explained by a model in which theta-form SV40 replication intermediates (Figure 7, I–III) break, giving rise to unidirectional forks that amplify the break by generating concatemers and branched concatemers [38], [39] (Figure 7, V, VI). Our data suggest that ATM kinase activity is crucial for the repair of one-ended replication-associated DSBs to reassemble bidirectional replication intermediates (Figure 7, VII) [49], [50], [51].

It is interesting to consider a possible role for unidirectional viral replication and its large concatemeric products in the tumorigenic activity of SV40, and more broadly of polyoma- and papillomaviruses. Concatemeric genomes of Merkel cell carcinoma virus and HPV are often integrated into human chromosomal DNA in tumors associated with these viruses [52], [53], [54]. The integration events and the consequences of long-term viral oncogene expression are primary risk factors for such cancers. It seems likely that in an infected cell under conditions of insufficient ATM activity, the level of viral concatemers would rise. With inadequate ATM activity, breaks in host chromosomal DNA would also be less frequently repaired through accurate, homology-dependent repair. Thus one can speculate that viral DNA concatemers generated under conditions of insufficient DNA damage signaling might be inaccurately joined with broken host chromatin, contributing to viral tumorigenesis [55].

### How does ATR signaling orchestrate SV40 replication fork convergence?

SV40 chromatin replication was highly sensitive to inhibition of ATR throughout a 48 h infection (Figures 5, S8). One consequence of ATR inhibition was that infected cells continued to cycle throughout infection, rather than arresting in late S phase where viral DNA replication would be favored [30]. However, the most prominent SV40 replication defect induced by ATRi was the tendency of converging replication forks to stall and break (Figures 6, 7, S9). Our data imply that after initiating replication at the viral origin, one replisome encounters an unknown replication block at variable positions in the viral genome (Figure 6F, S9, I and II, red triangle). Since the two sister Tag helicases need not remain coupled after initiation, they can proceed asynchronously as they replicate the viral genome bidirectionally [26], [56], [57], [58], [59]. Thus, the functional, unstalled replisome continues replication until it approaches the stalled fork (Figure 6F, III). We suggest that without ATR activity, the unstalled fork cannot converge with the stalled fork and breaks, yielding the pattern observed on the simple Y arc (Figure 6C, E, F, IV–VI). Consistent with this interpretation, fork convergence is well known to represent a slow step during unperturbed SV40 DNA replication in infected cells and to occur in a ∼1 kbp region around the BamHI site [60], [61], [62], suggesting that specialized host proteins and ATR-dependent modifications may be needed to complete replication.

Our observation that ATRi renders SV40 fork convergence prone to DNA breakage is reminiscent of common fragile sites in the human genome, which suffer gaps and breaks in Seckel Syndrome cells that express defective ATR alleles [63]. Thus SV40 and other small DNA tumor virus genomes may harbor a potential fragile site in the region where the two viral replication forks converge. Consistent with this speculation, C-terminal truncation of the polyomaviral T antigen encoded in the “fragile site” could render an integrated viral genome replication-defective and perhaps more tumorigenic [52], [64], [65], [66]. Similarly, the viral “fragile site” where replication forks converge would correspond to common viral genome breakpoints in integrated high risk papillomaviral genomes in cervical cancer [67], [68], [69].

## Materials and Methods

For details not described below, please refer to the online Supporting Methods (Protocol S1).

### Use of PIKK inhibitors

Ku-55933, kindly provided by Astra-Zeneca, was used as described [12], [29]. Importantly, Ku-55933 did not inhibit sixty off-target kinases. It specifically inhibits purified ATM with an IC50 of 12.9 nM, whereas it inhibits the related kinases mTOR and DNA-PK with IC_50_ values of 2500 nM and 9300 nM, respectively, *in vitro* [12]. Caffeine (Sigma) was dissolved to 24 mM in DMEM and used at a final concentration of 8 mM to inhibit ATM and ATR [40]. ATRi and Nu7441 were generous gifts from Dr. David Cortez. ATRi dissolved in DMSO at 5 mM was used at a final concentration of 5 µM [13]. ATRi selectively inhibits ATR with a K_i_ of 13 nM, whereas at least a 100-fold higher concentration is required *in vitro* to inhibit the related kinases ATM (K_i_ = 16000 nM), DNA-PK (K_i_ = 2200 nM), mTOR (K_i_ = 1000 nM), and PI3Kgamma (K_i_ = 3900 nM) [13]. Nu7441 was dissolved in DMSO to 2 mM and applied to cells at 1 µM [70], [71]. Nu7026 (EMD) was dissolved to 5 mM in DMSO and used at a final concentration of 10 µM [72].

DMEM containing inhibitor or solvent was added to cells 30 min prior to infection. At time zero, DMEM with inhibitor or solvent was removed, and fresh warm DMEM containing inhibitor or solvent and SV40 was added to cells. Cells were gently rocked every 15 min during the first 2 hpi. At 2 hpi, complete DMEM containing inhibitor or solvent was added to each dish of cells. At 20 hpi, medium was aspirated and cells were washed once with PBS to remove residual inhibitor or solvent. Fresh medium containing inhibitor or solvent was then added to cells and infections were allowed to proceed until the chosen endpoint. Solvent control treatments utilized the solvent concentration present in the inhibitor-treated medium.

### DNA isolation

Total intracellular DNA was prepared from infected and mock-infected cells. For each experiment, all samples were prepared from an equal number of cells. Cell pellets were resuspended in 0.4 ml of TE (10 mM Tris pH 8.0, 1 mM EDTA). SDS, RNase A, proteinase K, and Tris pH 7.5 were added to a final concentration of 0.4%, 0.2 mg/ml, 50 ug/ml and 100 mM, respectively, in a total volume of 0.5 ml. Following overnight digestion at 37°C, each sample was extracted twice with Tris-saturated phenol (pH 7.9) and once with 24∶1 chloroform: isoamyl alcohol. DNA was precipitated with sodium acetate and ethanol. DNA was allowed to dissolve in T0.1E (10 mM Tris pH 8.0, 0.1 mM EDTA) for 2 days, and then digested overnight at 37°C with 40 U of SacI-HF and XbaI (both from New England Biolabs). Digested DNA was re-precipitated and then dissolved in 50 µL of T0.1E per 2.5×10^5^ cells. Equal volumes of DNA were loaded on gels for southern blots unless otherwise indicated.

### Agarose gel electrophoresis

One-dimensional 0.7% agarose gels in 1× TAE were electrophoresed at 10 V/cm for 1.5 h. Neutral 2 d gel electrophoresis was performed as previously described [37] with the following modifications. The first dimension of the gel was electrophoresed at 1 V/cm through a 0.4% 1× TAE for 22 h. 1× TAE was found to enhance separation of D-loop arc (data not shown). The second dimension was electrophoresed at 5.5 V/cm through a 1.1% 1× TBE gel containing 0.5 ng/ml ethidium bromide for 5.5 h with circulation.

### Southern blotting analysis

Southern blotting was performed using radiolabeled probes for SV40 and BSC40 mitochondrial DNA as described [34]. A probe for human mitochondrial DNA was generated by PCR amplification (primers: U2OS Mito-F ACG CGA TAG CAT TGC GAG AC; U2OS Mito-R CTT TGG GGT TTG GTT GGT TCG), followed by random priming. Hybridized blots were visualized using a Typhoon Trio laser scanning imager (GE Healthcare) and quantified using ImageQuant 5.2 (GE Healthcare).

Bands or arcs corresponding to each DNA structure of interest were quantified and the value from a region of the blot without signal, e.g. Mock for SV40 probe, was subtracted as background. To compare the level of a DNA structure after a given treatment (e.g. DNA structure (% of Total DNA)), the total signals for the DNA were summed, and the signal of a discrete DNA structure (e.g. form I monomer) were divided by the total signal in the lane (e.g. [form I monomer signal]/[total signal in the lane]). To quantify variations in replication between treatments, all SV40 DNA signals were normalized using the respective mitochondrial DNA signal. Normalized signals were then divided by the normalized signal present in the infected solvent control to yield the DNA signal (% of DMSO).

The southern blot signals from an equal area of each arc in neutral 2 d gels were quantified (boxed areas in Figure 4C, D, E, F). Background signal in an area of equal size was subtracted, and the values for each arc were normalized to the value for the double Y (Figure 4H) or bubble arc (Figure 4I).

### Statistics

Statistics were performed in Microsoft Excel using the data analysis package. Prior to t-test, single factor ANOVA analysis was performed. If ANOVA resulted in p<0.5, a two sample t-test assuming unequal variances was performed. One-tailed p values from student's t test are denoted by the number of asterisk(s): * p<0.05 ** p<0.01 *** p<0.001 **** p<0.0001. All one tailed p values were generated by comparing data from SV40 infection in the presence of inhibitor to that from SV40 infection in the presence of DMSO. Bar graphs present the average of 3 to 4 independent experiments and error bars represent standard deviation.

## Supporting Information

Figure S1 **Viral replication centers co-localize with host DNA replication factors in SV40-infected BSC40 cells.** A–E. Merged images of chromatin-bound Tag and the indicated host DNA replication factors from mock- or SV40-infected BSC40 cells at 48 hpi. Top image for each replication protein is a mock-infected cell. The fluorescence intensity in arbitrary units (AU) along the line shown in the merged image is graphed in the right panel. Scale bars, 10 µm.
(TIF)

Figure S2 **Host DNA replication proteins co-localize with Tag in SV40-infected U2OS cells.** A–D. Representative images of chromatin-bound Tag and the indicated host DNA replication proteins from SV40-infected U2OS cells at 48 hpi. The fluorescence intensity in arbitrary units (AU) along the line shown in the merged image is graphed in the right panel. Scale bars, 10 µm.
(TIF)

Figure S3 **Aberrant DNA structures accumulate in ATM-inhibited SV40-infected U2OS cells.** A. Total DNA extracted at 48 hpi from SV40-infected BSC40 cells treated with Ku-55933 during the indicated phases of infection, as in Figure 2A, was analyzed by southern blot. Lanes 1–5: DNA digested with XbaI and SacI. Lanes 6–10: DNA digested with BglI. B. Southern blot of DNA replicated in SV40-infected U2OS cells in the presence of ATM inhibitor during the indicated phases of infection. C. Quantification of SV40 signal in monomeric forms and the whole sample in each lane, normalized to the corresponding signals in the DMSO solvent lane as in panel B. D and E. Fraction of signal in monomer forms (D) or in the indicated DNA structure (E) in DNA extracted at 48 hpi from cells treated with Ku-55933 during the indicated phases of infection as in panel B. Values in C–E represent the average of 3 to 4 independent experiments.
(EPS)

Figure S4 **Caffeine inhibits ATM and ATR activities in SV40-infected BSC40 cells.** A. BSC40 cells were treated with caffeine during the indicated phases of a 48 h SV40 infection. B and C. Western blots of cell lysates from SV40-infected BSC40 cells exposed to caffeine as depicted in (A).
(TIF)

Figure S5 **ATM and ATR inhibition increases aberrant DNA product accumulation.** A, E. Southern blots of total DNA extracted from BSC40 (A) or U2OS (E) cells treated with caffeine during phases of SV40 infection as described in Figure S4A. B, F. Quantification of signal in SV40 monomer forms or total DNA in caffeine-treated BSC40 (B) or U2OS (F) cells, normalized to that in DMEM solvent. C, D, G, H. SV40 signal in monomer forms (C and G) or aberrant DNA structures (D and H) accumulated in caffeine-treated BSC40 (C and D) or U2OS (G and H) cells, divided by the total SV40 DNA signal in respective lane. Bars in B–D and F–H represent the average of 3 to 4 independent experiments.
(EPS)

Figure S6 **DNA-PK activity is dispensable in unperturbed SV40 infection.** A. Experimental scheme for treatment of BSC40 cells with DNA PK inhibitors during phases of a 48 h SV40 infection. B., C. Southern blots of DNA extracted from BSC40 cells treated as in A with Nu7026 (B) or Nu7441(C). D. Quantification of SV40 replication products as in B, C.
(EPS)

Figure S7 **ATRi inhibits ATR activity in SV40-infected BSC40 cells.** A. Exposure of SV40-infected BSC40 cells to ATRi during defined phases of a 48 h infection. B. WST-1 viability assay of SV40-infected BSC40 cells treated with ATRi as described in A. Values were normalized to SV40-infected cells in the presence of DMSO. Error bars represent four independent experiments. C, D. Western blot of cell lysates from SV40-infected BSC40 cells exposed to ATRi as indicated.
(EPS)

Figure S8 **ATR is needed for efficient viral DNA replication in U2OS cells.** A. Southern blot analysis of total DNA from BSC40 cells treated with ATRi during the indicated phases of infection as in Figure S7A. Lanes 1–5: DNA digested with XbaI and SacI. Lanes 6–10: DNA digested with BglI. An equal amount of unit length SV40 DNA was loaded in each lane using the data in figure 5C using an equal number of cells. B. Total DNA from SV40-infected U2OS cells treated with ATRi as in Figure S7A was analyzed by southern blotting. C. Quantification of SV40 signal in total and monomeric SV40 DNA forms from infected U2OS cells treated with ATRi, normalized to the corresponding signals from infected cells treated with DMSO. D. Fraction of total SV40 signal in the indicated DNA structures in infected U2OS cells exposed to ATRi. Bars in graphs in C, D represent the average of 3 to 4 independent experiments.
(EPS)

Figure S9 **ATR inhibition results in replication fork stalling and breakage.** A. Diagrams of replication intermediates on a simple Y arc produced when ATR is inhibited. Cleavage sites are denoted as a colored vertical line: BglI (orange), BamHI (green). I. Replication begins at the origin and forks diverge bidirectionally to produce theta-form replication intermediates. II. Both replisomes progress unless a replication block (red triangle) is encountered, causing a fork to stall. III. The stalled replication fork is closest to orange BglI site (viral origin of replication). The functional replisome continues replication and converges with the stalled replication fork. IV. One-sided DSB forms at the replicating fork of the late Cairns intermediate shown in (III) as it approaches the stall site. V. Simple Y DNA structure generated by BglI or BamHI digestion of the broken late Cairns intermediate shown in (IV). VI. Diagram of the predicted outcome of the simple Y shown in panel (V) after neutral 2 d gel electrophoresis and southern blot analysis. The stall point on the simple Y arc (light green circle) corresponds to the simple Y in panel (V).
(EPS)

Protocol S1 **Supporting methods.**
(DOCX)
