# Supplementary figures and images for: ATM and ATR Activities Maintain Replication Fork Integrity during SV40 Chromatin Replication
Source: PLoS Pathog. 2013 Apr 4;9(4):e1003283. doi: 10.1371/journal.ppat.1003283 (PMC3617017; doi:10.1371/journal.ppat.1003283)

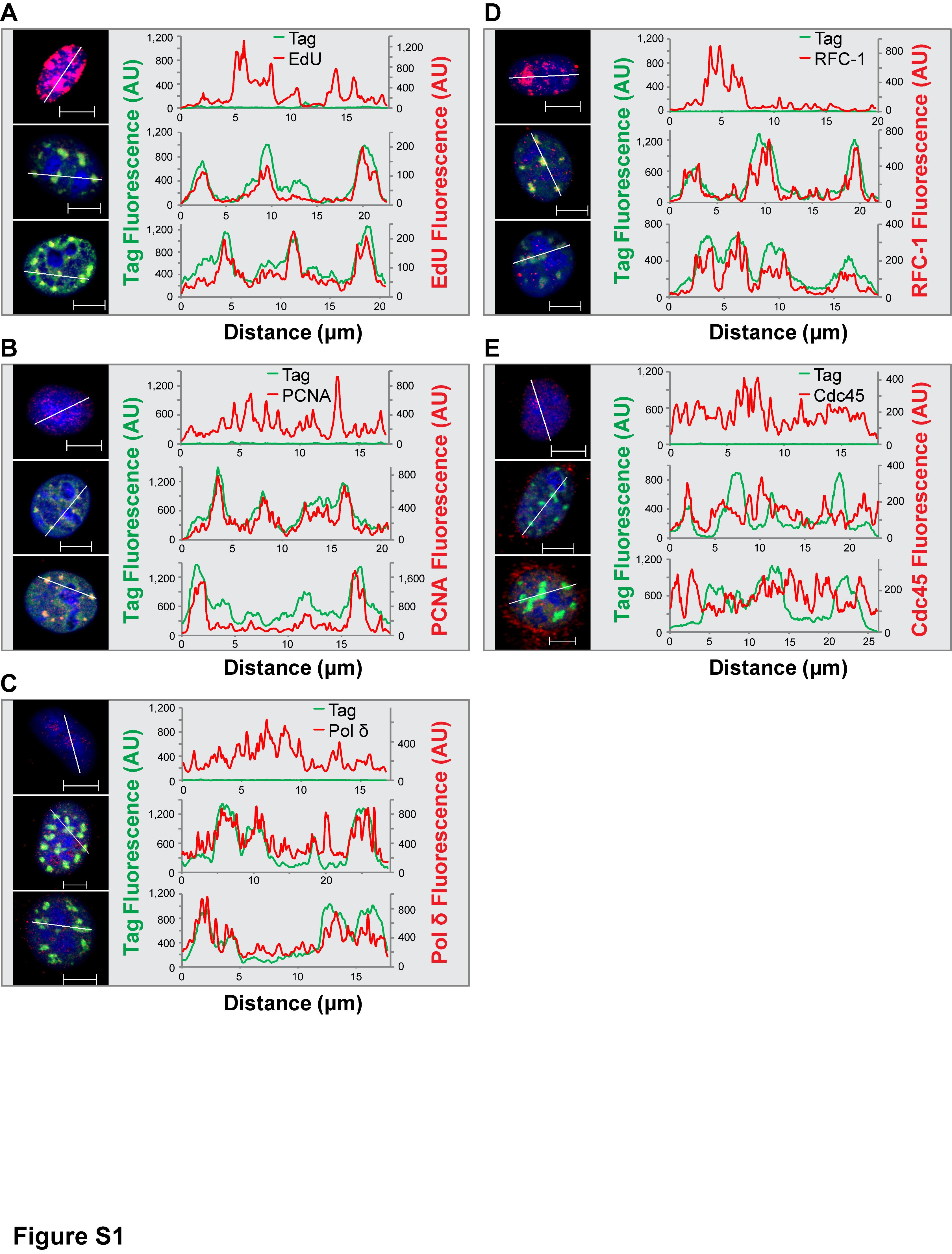

Supplement: Figure S1 — Viral replication centers co-localize with host DNA replication factors in SV40-infected BSC40 cells. A–E. Merged images of chromatin-bound Tag and the indicated host DNA replication factors from mock- or SV40-infected BSC40 cells at 48 hpi. Top image for each replication protein is a mock-infected cell. The fluorescence intensity in arbitrary units (AU) along the line shown in the merged image is graphed in the right panel. Scale bars, 10 µm. (TIF) [file ppat.1003283.s001.tif]

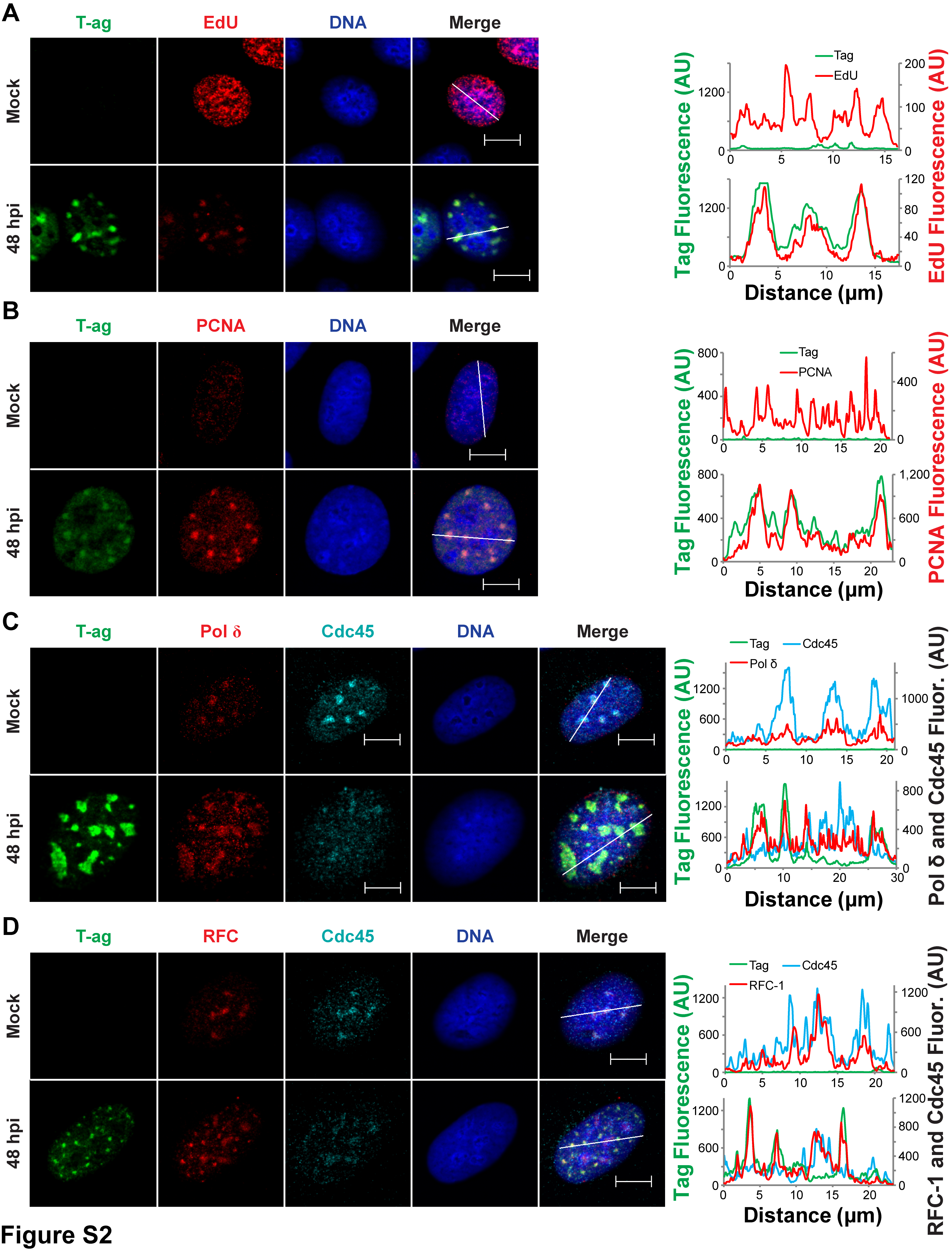

Supplement: Figure S2 — Host DNA replication proteins co-localize with Tag in SV40-infected U2OS cells. A–D. Representative images of chromatin-bound Tag and the indicated host DNA replication proteins from SV40-infected U2OS cells at 48 hpi. The fluorescence intensity in arbitrary units (AU) along the line shown in the merged image is graphed in the right panel. Scale bars, 10 µm. (TIF) [file ppat.1003283.s002.tif]

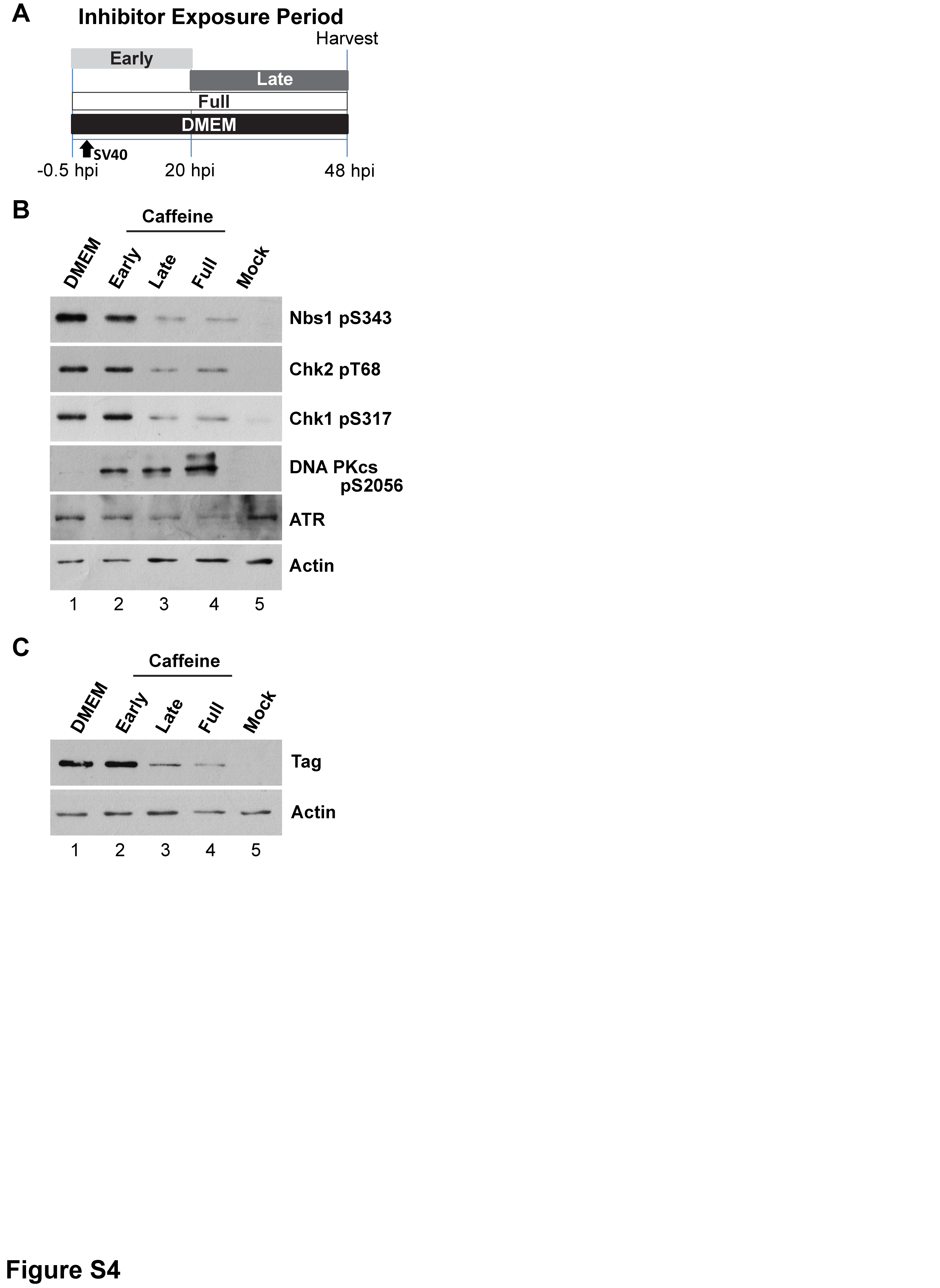

Supplement: Figure S4 — Caffeine inhibits ATM and ATR activities in SV40-infected BSC40 cells. A. BSC40 cells were treated with caffeine during the indicated phases of a 48 h SV40 infection. B and C. Western blots of cell lysates from SV40-infected BSC40 cells exposed to caffeine as depicted in (A). (TIF) [file ppat.1003283.s004.tif]
