# Supplementary material for: ATM and ATR Activities Maintain Replication Fork Integrity during SV40 Chromatin Replication
Source: PLoS Pathog. 2013 Apr 4;9(4):e1003283. doi: 10.1371/journal.ppat.1003283 (PMC3617017; doi:10.1371/journal.ppat.1003283)
Supplement: Protocol S1 — Supporting methods. (DOCX) [file ppat.1003283.s010.docx]

**Supporting Methods**

**Cells and SV40 infection**

BSC40 and U2OS cells were cultured in complete Dulbecco’s modified Eagle’s medium (DMEM supplemented with 10% fetal bovine serum) at 37°C with 5% CO_2_. For indirect immunofluorescence, cells were grown on an 18 x 18 mm glass cover slip (Fisher) in 35 mm dishes. Prior to SV40 infection, equal numbers of cells were plated into dishes. A duplicate dish was counted to determine total cell number, and cells were infected with DMEM containing SV40 at a multiplicity of infection of 10 (BSC40) or 115 (U2OS) focus forming units/cell. Mock infections were performed in parallel, by using, an equal volume of DMEM lacking virus.

**WST-1 viability assay**

BSC40 cells were plated into wells of a 96 well plate and treated with ATRi as described in materials and methods under the inhibitors and treatments subheading. At 48 hpi, WST-1 assay was performed according to manufacturer protocol (Roche). To obtain the viability of ATRi treated SV40-infected or mock infected cells compared to SV40-infected cells in the presence of DMSO (Viability (% of DMSO), the OD of mock or SV40-infected cells in the presence/ absence of ATRi was divided by the OD generated from SV40 infected cells in the presence of DMSO.

**Plasmids and transfection**

pMini SV40-wt and pMini SV40-D474N were as previously described [1]. The In-1 mutation [2,3] was introduced by standard site-directed mutagenesis into pMini SV40 wt and verified by DNA sequencing. pMini SV40-In-1 contains a single G-C base pair insertion at position 1 of the SV40 genome, abrogating the BglI site. For transfection, 2 ug of supercoiled pMini SV40-wt or mutant plasmid was transfected into a semi-confluent 35 mm plate of BSC40 cells using Fugene HD (Roche) as per manufacturer protocol.

**Low molecular weight DNA extraction**

Equal numbers of cells transfected with pMini-SV40 plasmid were resuspended in 383 μL T10E (20 mM Tris pH 7.5 and 10 mM EDTA). SDS and RNAseA were added to a final concentration of 0.6% and 0.1 mg/mL, respectively. To ensure equal cell lysis, tubes were inverted ten times prior to addition of NaCl to a final concentration of 1M to bring the final volume to 500 μL. High molecular weight DNA was allowed to precipitate overnight at 4°C. DNA was spun at 17000xg for 30 min to pellet host genomic DNA. Equal volumes of supernatant were kept and twice extracted with saturated phenol (pH 7.9) followed by one extraction with 24:1 chloroform: isoamyl alcohol. The resulting supernatants were precipitated with sodium acetate and ethanol. DNA pellets were dissolved in T0.1E overnight and then digested with 20 U of DpnI overnight to digest unreplicated DNA. Following re-precipitation of DNA with sodium acetate and ethanol, DNA was dissolved in 25 μL of T0.1E per 5x10^5^ cells. Equal volumes of DNA were loaded on gels for southern blots.

**Western blots**

Whole cell lysates were prepared as previously described [1], except that 1 mM NaVO_4_ and 5 mM NaF were added during lysis to inhibit phosphatases. Samples containing 10 ug of protein were analyzed by SDS-PAGE and western blotting. The following antibodies were used: anti-Tag (Pab101, [4]), anti-actin (I-19, Santa Cruz), anti-Chk1 (G-4, Santa Cruz), anti-Chk1 pS317 (Cell Signaling), anti-Chk2 pT68 (Y171, Epitomics), anti-Chk2 (EPR4325, Epitomics), anti-Nbs1 pS343 (EP178, Epitomics), anti-ATR (N-19, Santa Cruz), and anti-DNA PK pS2056 (EPR5670, Epitomics).

**Immunofluorescence microscopy**

To visualize chromatin-associated proteins, soluble proteins were pre-extracted from cells, followed by fixation and immunostaining as described [5]. For EdU labeling of DNA, 10 uM EdU nucleoside in complete DMEM was added to cells for 5 min. At 48 hpi, cover slips were processed for immunostaining and click reaction according to the manufacturer’s protocol (Invitrogen). All micrographs were taken using an AxioObserver Z1 (Zeiss) equipped with a 63x Plan Apochromat (NA 1.4) oil objective (Zeiss) and an apotome (0.6 μm z slice) (Zeiss). To quantify Tag staining patterns in SV40-infected BSC40 cells treated with Ku-55933 as in Figure 2A, aberrant and normal Tag staining patterns from 3 independent experiments, each with at least 50 cells, were counted and the average values were graphed.

Primary antibodies used for immunostaining were anti-Tag (Pab101), rabbit anti-Tag (in house), anti-γH2AX (JBW301, Millipore), anti-Cdc45 [6], anti-PCNA (PC10, Santa Cruz), anti-polymerase δ p125 (C-2, Santa Cruz), and anti-RFC1 (H-300, Santa Cruz). Secondary antibodies used for immunostaining were anti-mouse conjugated to Alexa Fluor 488 (Invitrogen), anti-rabbit conjugated to Alexa Fluor 555 (Invitrogen), and anti-rat conjugated to Dylight 649 (Jackson ImmunoResearch).

**References**

1. Zhou B, Arnett DR, Yu X, Brewster A, Sowd GA, et al. (2012) Structural basis for the interaction of a hexameric replicative helicase with the regulatory subunit of human DNA polymerase alpha-primase. J Biol Chem 287: 26854-26866.

2. Cohen GL, Wright PJ, DeLucia AL, Lewton BA, Anderson ME, et al. (1984) Critical spatial requirement within the origin of simian virus 40 DNA replication. J Virol 51: 91-96.

3. Virshup DM, Russo AA, Kelly TJ (1992) Mechanism of activation of simian virus 40 DNA replication by protein phosphatase 2A. Mol Cell Biol 12: 4883-4895.

4. Deppert W, Gurney EG, Harrison RO (1981) Monoclonal antibodies against simian virus 40 tumor antigens: analysis of antigenic binding sites, using adenovirus type 2-simian virus 40 hybrid viruses. J Virol 37: 478-482.

5. Zhao X, Madden-Fuentes RJ, Lou BX, Pipas JM, Gerhardt J, et al. (2008) Ataxia telangiectasia-mutated damage-signaling kinase- and proteasome-dependent destruction of Mre11-Rad50-Nbs1 subunits in Simian virus 40-infected primate cells. J Virol 82: 5316-5328.

6. Bauerschmidt C, Pollok S, Kremmer E, Nasheuer HP, Grosse F (2007) Interactions of human Cdc45 with the Mcm2-7 complex, the GINS complex, and DNA polymerases delta and epsilon during S phase. Genes Cells 12: 745-758.
